# Supplementary material for: DIA-Based Proteomic Analysis Reveals MYOZ2 as a Key Protein Affecting Muscle Growth and Development in Hybrid Sheep
Source: Int J Mol Sci. 2024 Mar 4;25(5):2975. doi: 10.3390/ijms25052975 (PMC10931989; doi:10.3390/ijms25052975)
Supplement: Supplementary file 1 [file ijms-25-02975-s001.zip › Figure S1-S4.pdf]

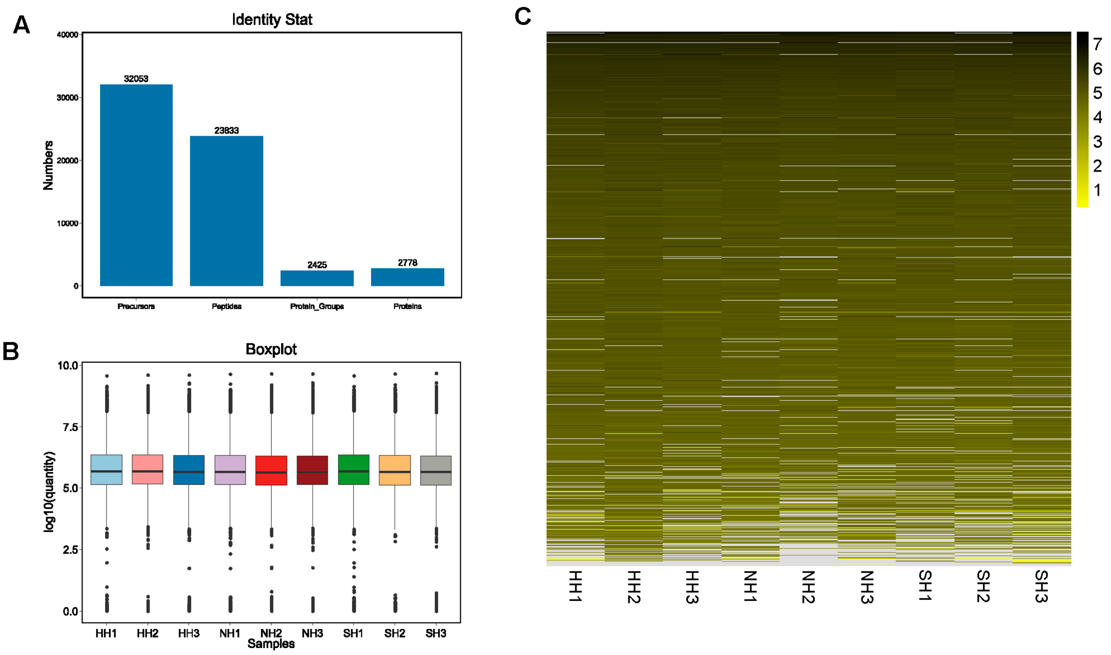

Figure S1. Protein quantitative normalization treatment

(A) Protein identification statistics. According to the filtration standard of FDR ≤ 0.01, the number of proteins and peptides that meet the requirements were screened.

(B) Normalization box plot. The local normalization method in Pulsar was used to normalize the peak intensity of the overall sample.

(C) Protein quantitation heatmap. The average of the peak areas of the first 3 MS1 peptides with an FDR of less than 1.0% was screened for protein quantification. log10 treatment, the darker the color, the higher the expression, the more yellow the color, the lower the expression, and the white color, the more the sample does not have the protein.

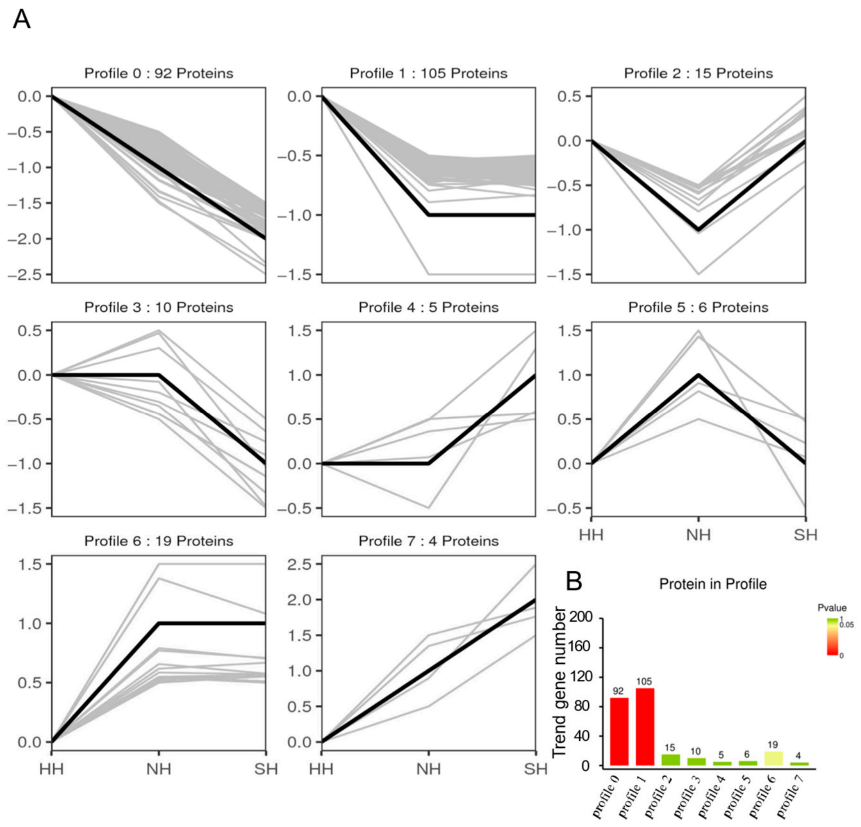

Figure S2. Trend Analysis of Differential Genes

(A) Trend pattern diagram. The black line represents the trend line and the gray line represents each gene.

(B) Trend gene number.

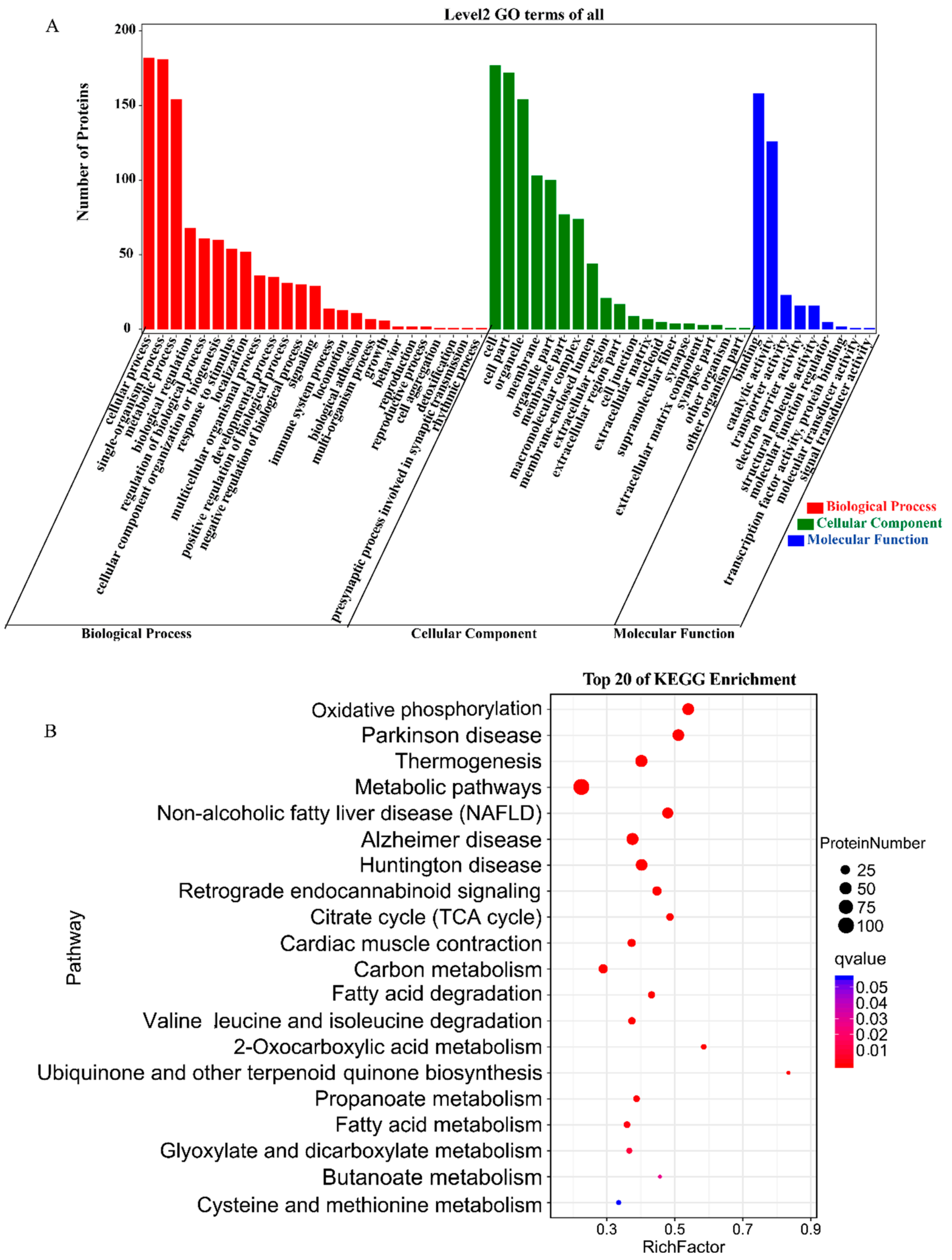

Figure S3. Functional enrichment analysis

(A) GO enrichment classification bar chart. (B)KEGG enrichment bubble diagram.

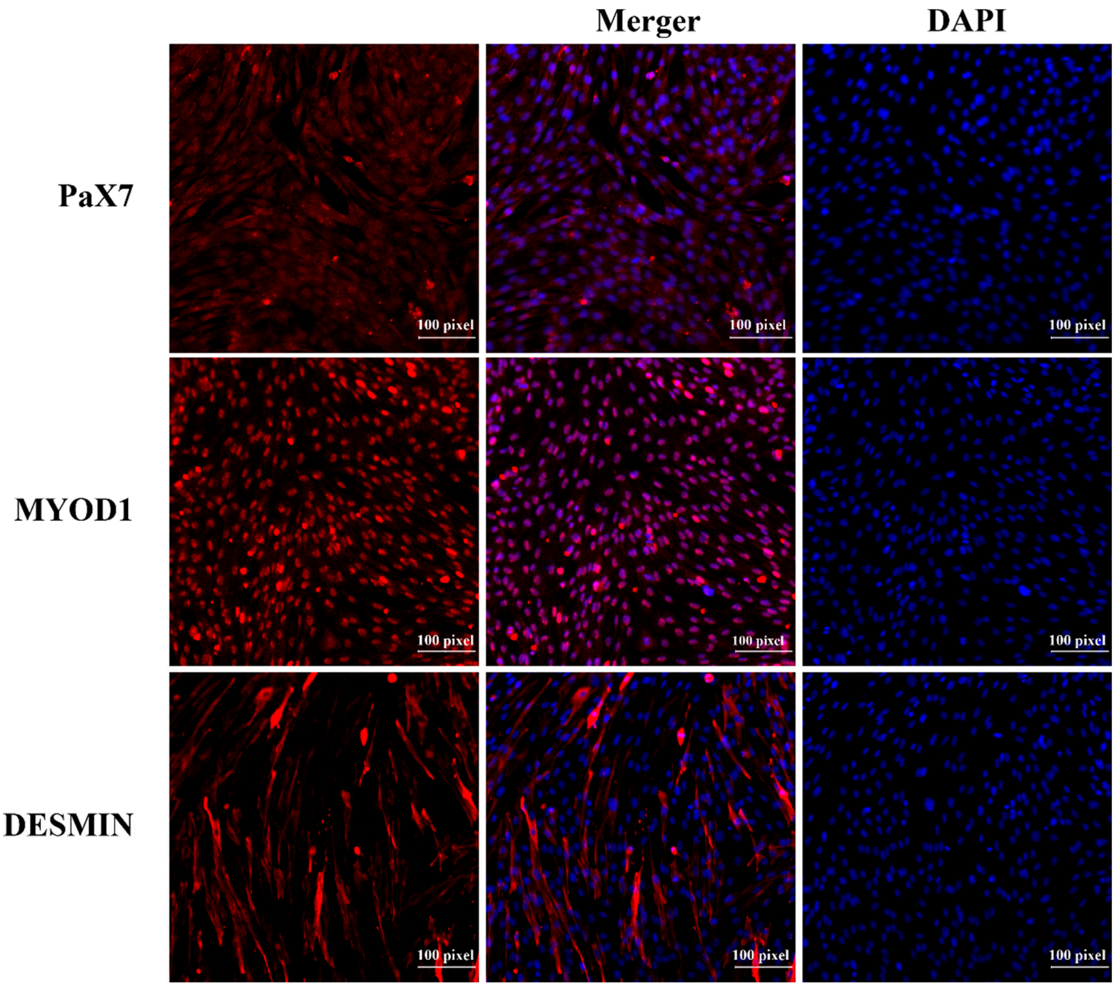

Figure S4. IF test of myoblast purity
