# Supplementary material for: DIA-Based Proteomic Analysis Reveals MYOZ2 as a Key Protein Affecting Muscle Growth and Development in Hybrid Sheep
Source: Int J Mol Sci. 2024 Mar 4;25(5):2975. doi: 10.3390/ijms25052975 (PMC10931989; doi:10.3390/ijms25052975)
Supplement: Supplementary file 1 [file ijms-25-02975-s001.zip › Table S10.pdf]

MYOZ2 overexpression vector

Gene name	Gene ID	Gene length （bp）	Carrier name
sheep MYOZ2	>HM628579.1:79-873 Ovis aries myozenin 2 (MYOZ2) mRNA, complete cds	795	pEX-3(pGCMV/MCS/Neo)
