# Supplementary material for: DIA-Based Proteomic Analysis Reveals MYOZ2 as a Key Protein Affecting Muscle Growth and Development in Hybrid Sheep
Source: Int J Mol Sci. 2024 Mar 4;25(5):2975. doi: 10.3390/ijms25052975 (PMC10931989; doi:10.3390/ijms25052975)
Supplement: Supplementary file 1 [file ijms-25-02975-s001.zip › Table S11.pdf]

MYOZ2 interferes with the vector

Carrier type	Gene name	Target site	Sequence（5'-3')	Gene ID
pGPU6/GFP/Neo	MYOZ2-Ovis aries	758	CTGGCAGACGGTCCTTTAATA	HM628579.1
