# Supplementary material for: DIA-Based Proteomic Analysis Reveals MYOZ2 as a Key Protein Affecting Muscle Growth and Development in Hybrid Sheep
Source: Int J Mol Sci. 2024 Mar 4;25(5):2975. doi: 10.3390/ijms25052975 (PMC10931989; doi:10.3390/ijms25052975)
Supplement: Supplementary file 1 [file ijms-25-02975-s001.zip › Table S12.pdf]

Primer information

Gene name	Gene ID	F	R	Gene length
MYOZ2	HM628579.1	CAGGCCATTAGCAAT GATCCG	TGGCAACTCTGTTAAA GCTC	109bp
MyoD	AF184163.1	CCTGCCTGTTCTGCGA GTTCC	ATGCCGCAGTCCAGG TCACA	157bp
MyoG	GU550517.1	ATGCAGCTCCCATAGT GCCTC	CCACTGTGATGCTGTC CACGA	149bp
Pax7	XM_027965644 .2	CGTGGAGTCAGAACC CGACCT	CGCCAGCTCCTCTCGG GTGT	142bp
MYF5	KT899092.1	ACCAGGCTTTCGACA CGCTCA	GCCATCAGAGCAACT TGAGGT	200bp
