# Supplementary material for: DIA-Based Proteomic Analysis Reveals MYOZ2 as a Key Protein Affecting Muscle Growth and Development in Hybrid Sheep
Source: Int J Mol Sci. 2024 Mar 4;25(5):2975. doi: 10.3390/ijms25052975 (PMC10931989; doi:10.3390/ijms25052975)
Supplement: Supplementary file 1 [file ijms-25-02975-s001.zip › Table S6.pdf]

List of instruments

Equipment	Manufacturers	Model
Hand Centrifuge	IKA	Mini G
Manual single-channel pipette	eppendorf	100-1000μl
Manual single-channel pipette	eppendorf	20-200μl
Manual single-channel pipette	eppendorf	10-100μl
Manual single-channel pipette	eppendorf	2-20μl
Manual single-channel pipette	eppendorf	0.5-10μl
Manual single-channel pipette	eppendorf	0.1-2.5μl
Vortex oscillator	Kylin-Bell	FC5306
4 °C refrigerator	Sharp	BCD-282WT
-20℃ refrigerator	SAMSUNG	RS542NCAEWW/SC
-80℃ refrigerator	Thermofisher	
Ice machine	Panasonic	SIM-F140ADL
Ultrasonic Cell Breaker	Thermofisher	
4 °C refrigerated centrifuge	Thermofisher	HERAEUS FRESCO 21
Analytical balance	Sartorius	0.1mg
High-throughput tissue fragmentation instrument	MP Biomedicals	FastPrep-24™5G
Enzyme calibration	Bio Tek	Synergy™2
Electrophoresis apparatus	Bio-Rad	PowerPac™ Universal
Vertical electrophoresis tank	Thermofisher	Mini Gel Tank
Constant temperature mixer	Eppendorf	Thermo Mixer
Bleaching shaker	HUAIDA	ZD-9560
Microwave oven	Midea	M1-L213B white
UltiMate3000	Thermofisher	UltiMate3000
Desktop rapid centrifugal concentration dryer	Thermofisher	SAVANT SPD1010
