# Supplementary material for: DIA-Based Proteomic Analysis Reveals MYOZ2 as a Key Protein Affecting Muscle Growth and Development in Hybrid Sheep
Source: Int J Mol Sci. 2024 Mar 4;25(5):2975. doi: 10.3390/ijms25052975 (PMC10931989; doi:10.3390/ijms25052975)
Supplement: Supplementary file 1 [file ijms-25-02975-s001.zip › Table S7.pdf]

Data analysis website

Software or database	Function specification	Version	Parameter	Link
Pfam	Pfam database	v32	default	http://pfam.xfam.org/
Plant TFdb	Prediction of Plant Transcription Factors	v4.0	default	http://planttfdb.cbi.pku.edu.cn/
Animal TFdb	Animal transcription factor prediction	v2.0	default	http://bioinfo.life.hust.edu.cn/AnimalTFDB
WoLFPSort	subcellular localization	--		https://www.genscript.com/wolf-psort.html
GSEA	GSEA analysis	v2.2.4	default	http://software.broadinstitute.org/gsea/index.jsp
