# Supplementary material for: DIA-Based Proteomic Analysis Reveals MYOZ2 as a Key Protein Affecting Muscle Growth and Development in Hybrid Sheep
Source: Int J Mol Sci. 2024 Mar 4;25(5):2975. doi: 10.3390/ijms25052975 (PMC10931989; doi:10.3390/ijms25052975)
Supplement: Supplementary file 1 [file ijms-25-02975-s001.zip › Table S9.pdf]

The antibodies used in this study for IF

Target protein	Source	Host	IF
PEX7	Proteintech	Rabbit	1:200
MYOD	Proteintech	Rabbit	1:200
DESMIN	Proteintech	Rabbit	1:200
MYH3	Bioss	Rabbit	1:200
Cy3	Bioss	Rabbit	1:80
